# Impact of Physical Activity Differences Due to COVID-19 Pandemic Lockdown on Non-Alcoholic Fatty Liver Parameters in Adults with Metabolic Syndrome

**DOI:** 10.3390/nu14122370

**Published:** 2022-06-08

**Authors:** Catalina M. Mascaró, Cristina Bouzas, Sofía Montemayor, Silvia García, David Mateos, Miguel Casares, Cristina Gómez, Lucía Ugarriza, Pere-Antoni Borràs, J. Alfredo Martínez, Josep A. Tur

**Affiliations:** 1 Research Group on Community Nutrition and Oxidative Stress, University of the Balearic Islands-IUNICS, 07122 Palma de Mallorca, Spain; c.mascaro@uib.es (C.M.M.); cristina.bouzas@uib.es (C.B.); sofiamf16@gmail.com (S.M.); silvia.garcia@uib.es (S.G.); davidfrom13@gmail.com (D.M.); cristina.gomez@ssib.es (C.G.); luciaugarriza@gmail.com (L.U.); 2 Health Institute of the Balearic Islands (IDISBA), 07120 Palma de Mallorca, Spain; 3 CIBEROBN (Physiopathology of Obesity and Nutrition CB12/03/30038), Instituto de Salud Carlos III (ISCIII), 28029 Madrid, Spain; 4 Radiodiagnosis Service, Red Asistencial Juaneda, 07011 Palma de Mallorca, Spain; casaresmiguel@gmail.com; 5 Clinical Analysis Service, University Hospital Son Espases, 07120 Palma de Mallorca, Spain; 6 Camp Redó Primary Health Care Center, 07010 Palma de Mallorca, Spain; 7 Area of Physical Education and Sports, Department of Pedagogy and Specific Didactics, University of the Balearic Islands, 07122 Palma de Mallorca, Spain; pa-borras@uib.es; 8 Cardiometabolics Precision Nutrition Program, IMDEA Food, CEI UAM-CSIC, 28049 Madrid, Spain; jalfredo.martinez@imdea.org; 9 Department of Nutrition, Food Sciences, and Physiology, University of Navarra, 31008 Pamplona, Spain

**Keywords:** Mediterranean diet, physical activity, Mediterranean lifestyle, non-alcoholic fatty liver disease, metabolic syndrome, fitness, COVID-19 lockdown

## Abstract

Background: A Mediterranean lifestyle with a Mediterranean diet and regular physical activity (PA) improves metabolic syndrome (MetS) characteristics and non-alcoholic fatty liver disease (NAFLD). The COVID-19 pandemic stopped healthy habits and increased NAFLD progression. Objectives: To assess how PA differences due to COVID-19 lockdown affected NAFLD parameters in adults with MetS. Design: Longitudinal 2-year analysis of data obtained between COVID-19 pre- and post-lockdown in a parallel-group randomized trial (n = 57, aged 40–60 years old, with MetS and NAFLD). Methods: NAFLD status and related parameters were assessed by magnetic resonance imaging (MRI), blood collection analysis and related indexes. PA and fitness status were assessed by an Alpha-Fit test battery, accelerometers, validated Minnesota questionnaire and functional fitness score. During lockdown, study personnel telephoned patients to motivate them. Participants were grouped according to PA levels. Results: The low PA group improved its fitness score tests (0.2) after lockdown more than the medium PA group, and it decreased its sedentary activity (−48.7 min/day), increased light (20.9 min/day) and moderate (32.3 min/day) PA intensities and improved sleep efficiency (0.6%) in comparison with the medium and high PA groups. The high PA group increased its steps per day more than the other groups. The low PA group was the only group that decreased its gamma glutamyl transferase (GGT) levels (−17.0 U/L). All groups increased their fatty liver index (FLI) after lockdown, but the medium PA group increased its FLI more than the low PA group. Participants in the high PA group decreased their HDL-cholesterol levels more than participants in the medium PA group (−0.4 mg/dL). Conclusions: Stopping regular PA together with an unhealthy lifestyle leads to a worsening of MetS and NAFLD. COVID-19 lockdown induced a decrease in PA in more active people, but inactive people increased their PA levels. Motivation seemed to be very important during lockdown.

## 1. Introduction

Non-alcoholic fatty liver disease (NAFLD) is the most common hepatic disease in Western countries. Currently, it affects up to 30% of the population and it is also considered a metabolic syndrome (MetS) manifestation [1,2]. NAFLD results in the deposition of fat in hepatocytes and it can progress to non-alcoholic steatohepatitis (NASH), fibrosis, cirrhosis and carcinoma [3,4] in the absence of excessive drug and/or alcohol consumption [5]. There is no pharmacological treatment for NAFLD, so the best existing treatment is lifestyle modification with a Mediterranean diet and physical activity (PA), as well as the treatment of each of the metabolic complications [6,7].

A Mediterranean diet is rich in unsaturated fatty acids, antioxidants and fiber; in addition, it provides a lower intake of saturated fats and animal proteins. This dietary composition prevents and/or improves type 2 diabetes mellitus, MetS and cardiovascular pathologies [8]. Besides that, PA decreases fatty liver content in people with NAFLD and improves MetS parameters (i.e., obesity, hypertension, high triglycerides and low plasma high-density lipoprotein (HDL) cholesterol levels) [9].

The COVID-19 pandemic increased sedentary activity and decreased regular PA, increased obesity and adversely affected mental health [10]. During lockdown, metabolic risk increased in people with NAFLD because adherence to a Mediterranean diet and regular PA decreased, so that in most cases, an increase in body weight and the progression of a fatty liver were observed [11].

There is considerable scientific evidence for health benefits of regular PA practice. In addition, more studies have demonstrated the benefits of PA and a Mediterranean diet on NAFLD. However, there is very little evidence assessing the impact of stopping a healthy lifestyle and regular PA on the different parameters of PA and NAFLD. Fortunately, COVID-19 has made it possible to assess this impact in the present manuscript. The aim of the current study was to assess how PA differences due to COVID-19 lockdown affected NAFLD parameters in adults with MetS.

## 2. Methods

### 2.1. Design

This research is a longitudinal analysis of pre- and post-COVID-19 lockdown data from a 2-year, randomized, parallel group trial. This trial was designed to assess the effect of changes in lifestyle with a Mediterranean diet and regular PA on preventing and/or reversing NAFLD in adults with MetS. Further details of the study protocol can be found at ClinicalTrials.gov (https://clinicaltrials.gov/ct2/show/NCT04442620; accessed on 25 February 2022) where the trial was registered in 2020 with number NCT04442620 [12]. 

During the development of this study, the COVID-19 pandemic and subsequent lockdown appeared, so the authors decided to study the impact of stopping a healthy lifestyle. Information and study variables remained the same, but the study time focused on the lockdown period and study groups were classified according to differences in METs/day measured by an accelerometer pre–post COVID-19 lockdown.

### 2.2. Subjects

Eligible participants were men and women aged 40–60 years with a body mass index (BMI) between 27 and 40 kg/m^2^ meeting at least three criteria for MetS according to the International Diabetes Federation (IDF) [13]), and with a diagnosis of NAFLD by magnetic resonance imaging (MRI) (Signa Explorer 1.5 T, General Electric Healthcare, Chicago, IL, USA) [14]. Exclusion criteria are available elsewhere [12]. We contacted 143 subjects for an initial screening between June of 2018 and January of 2020 in Mallorca (Balearic Islands), and a total of 57 subjects were eligible for the study. A total of 86 subjects were excluded because they did not meet inclusion criteria or declined their participation. 

All participants were informed about the study before participating and they provided written consent. The study protocol and all procedures were performed and approved according to the ethical standards of the Declaration of Helsinki and Ethics Committee of Research of Balearic Islands (ref. IB 2251/14 PI).

### 2.3. Intervention Groups

Selected individuals were randomized by gender (men/women), type 2 diabetes mellitus (yes/no) and grade of steatosis into one of the three following groups (1:1:1 ratio): (1) Conventional diet (CD) group, based on the recommendations of the American Association for the Study of Liver Diseases (AASLD) [15]. PA recommendation was walking a minimum 10,000 steps per day. (2) Mediterranean diet-high meal frequency (MD-HMF) group, based on a RESMENA diet [16] with 7 meals a day. PA recommendation was walking minimum 10,000 steps per day. (3) Mediterranean diet-physical activity (MD-PA) group, based on a Mediterranean diet [17] with 5 meals a day. PA recommendation was performing 35 min interval training sessions (5 min warm-up, 20 min training, and 10 min breathing and stretching) 3 days per week (2 days with an instructor and 1 day a remote session). The 20 min training consisted of 5 moderate intensity tests, with recovery time in between each test. Each week, the tests changed to make the sessions more dynamic. Remote sessions were sent to participants by email and/or WhatsApp, and the PA was personalized to suit individual conditions. Aerobic PA in this group was equivalent to 10,000 steps per day referent to caloric expenditure, i.e., 400 kcal/70 kg. 

The above PA recommendations were before lockdown. During COVID-19 confinement, all training sessions were cancelled, and people walking 10,000 steps daily had their route interrupted. Study personnel contacted each of the participants by telephone and encouraged them to continue with the respective interventions. Regarding to PA, subjects in the CD and MD-HMF groups were encouraged to complete a minimum of 10,000 steps per day walking around their home or garden (depending on the possibilities). Subjects in MD-PA group were informed to continue with all sessions remotely at least 3 days a week. Through email and WhatsApp, instructors sent them a large number of videos, photos and enough material to continue with sessions without an instructor’s presence, and even some video calls were made to follow the sessions remotely. In addition, a WhatsApp group was created so that they could exchange other ideas and motivate each other. 

In the current paper, data analyzed comprised the latest available before lockdown and the first collected after lockdown, provided they were recorded within a period of four months before/after lockdown start/end. The lockdown period was 3 months, so pre-lockdown data were taken within a range of 4 months before, and post-lockdown data were also taken within a range of 4 months after.

### 2.4. Description of the Lockdown

The Spanish government ordered home lockdown for 3 months. Adults were forced to work from home and children had to study from home (schools were closed). Since it was a sudden order, no adaptation procedures were allowed. The situation forced some companies to shut down, however the government provided financial aid for affected workers. As people were not allowed to leave their houses, physical activity levels changed during confinement. Moreover, as a result of the increase in free time and boredom, people started to cook more, especially bakery [18,19]. 

### 2.5. Blood Collection Analysis

Biochemical analysis was performed on participants’ blood samples, which were collected from an antecubital vein after twelve hours overnight fast. All studied parameters were quantified by standard enzymatic methods [20]. These collections assessed aspartate aminotransferase (AST), alanine aminotransferase (ALT), gamma-glutamyl transferase (GGT), triglycerides, glucose and HDL-cholesterol.

In the original study, samples were to be collected at baseline, 6 months, 1 year and 2 years. For the present manuscript, samples used for the analysis were those closest to pre- and post-lockdown. 

### 2.6. NAFLD Diagnosis

As previously mentioned, patients had to undergo an MRI (Signa Explorer 1.5T, General Electric Healthcare, Chicago, IL, USA) [14] to confirm a diagnosis of NAFLD, and later, to measure progression in the amount of liver fat (expressed as a percentage). 

Again, in the original study, the MRI was to be performed at baseline, 6 months, 1 year and 2 years; for the present manuscript, the MRI results used for the analysis were those closest to pre- and post-lockdown.

### 2.7. NAFLD Related Indexes

In the current manuscript, two indexes were calculated to describe fatty liver status based on other measurements from non-invasive methods. Fatty liver index (FLI) allows individuals with NAFLD to be identified and to predict new cases. Patients with an FLI <30 were considered not to have NAFLD, whereas an FLI ≥60 indicated NAFLD [21]. To calculate FLI, triglycerides and GGT levels, BMI and waist circumference are entered into a previously defined formula [21]. Hepatic steatosis index (HSI) is a screening tool that identifies the presence of NAFLD. Patients with an HSI <30 were considered to not have NAFLD; HSI = 30–36 was considered an intermediate status; and HSI ≥36 indicated the presence of NAFLD [22]. To calculate HSI, ALT and AST levels, BMI, and the patient’s sex and diabetic status were entered into a formula previously published [22].

### 2.8. Physical Activity Assessment

PA was evaluated by three methods in order to achieve greater accuracy. Participants completed tests from “Fitness for Health: The Alpha-Fit Test Battery for Adults aged 18–69” [23] and the Chester step test [24]. The Alpha-Fit manual was used to assess postural control (one-leg balance test), static manual muscular strength (standing hand grip test), sitting manual muscular strength (sitting hand grip test), resistance of lower extremities (jump-and-reach test) and resistance of upper extremities and trunk (modified push-up test), as previously described [9]. The Chester step test was used to assess aerobic capacity and was calculated using software [25]. All mentioned tests were performed on the same day and in the same order that they were presented by trained personal. The best mark of each test was taken to compare with normal range of scores according to gender and age [23]. 

In addition, participants were asked to complete the Spanish version of the Minnesota Leisure Time Physical Activity Questionnaire, reporting their leisure and household activity as mean weekly time. Resulting energy expenditure was expressed as the metabolic equivalent of task (MET)·minute/week [26,27]. 

All patients had to wear an accelerometer (ActiGraph wGT3X-B; ActiGraph LLC, Pensacola, FL, USA) on their left wrist for one week. It measured sedentary, light, moderate and vigorous intensities of PA (the last category does not appear in the manuscript because nobody registered vigorous PA), weekly steps and sleep efficiency. Measured energy expenditure was expressed as metabolic equivalent of task (MET)·minute/week. All participants were then classified into three groups according to PA: Low PA/Medium PA/High PA. A grouping variable was calculated from the difference in METs/day measured by the accelerometer pre-post COVID-19 lockdown followed by tertiles for this pre-post-lockdown PA difference.

### 2.9. Functional Fitness Score

A previously defined functional fitness score was calculated [28] from a normal range for one-leg balance, standing hand grip, jump-and-reach and modified push-up test with a median defined as sex. Scores below the normal range and the median by sex were awarded 0 points. Scores above the normal range and the median by sex and were awarded 1 point. The best functional fitness score was obtained from 1 point for each test, i.e., a final score of 4 points. This fitness score described the physical function of the participants as a whole and were used to recommend alternatives to PA and better health promotion [28]. 

### 2.10. Other Health Outcomes

Information related to sociodemographic characteristics were obtained by questionnaires: age, gender, education level (years), marital status (single; married/coupled; divorced/separated/widow), socioeconomic status according to job (low, medium/high), smoking habit (no, ≥1 cigarette/day) and alcohol consumption (no, <7 drinks/week, ≥7 drinks/week). 

Blood pressure was measured using a validated semi-automatic oscillometer (Omron HEM-705CP, Omron Healthcare Inc., Lake Forest, IL, USA) [20] and waist circumference was taken [20]. During lockdown, study personnel had telephonic contact with patients to help and motivate them to follow the diet and perform PA.

### 2.11. Statistics

Analyses were performed with the SPSS statistical software package version 27.0 (SPSSS Inc., Chicago, IL, USA). Data are shown as mean and standard deviation (SD), whereas prevalence was expressed in sample size and percentage. Differences in prevalence among groups was tested using χ^2^ (all *p* values were two-tailed). The normal distribution for continuous data was assessed with Kolmogorov–Smirnov. All variables with non-normal distribution were log-transformed before analysis; however, this data is presented as untransformed data in tables for easier interpretation. 

Analysis of sociodemographic characteristics from baseline were tested with a one-way ANOVA and Bonferroni’s post-hoc analysis for continuous variables and χ^2^ for prevalence. On the other hand, paired sample t-tests were used to compare changes in PA and NAFLD parameters from COVID-19 pre-lockdown to COVID-19 post-lockdown within each of the three PA difference groups. A generalized linear model was used to assess changes in PA and NAFLD parameters during COVID-19 lockdown according to the three PA difference groups (between-group). The impact of the COVID-19 lockdown and PA differences was examined by using a repeated-measures ANCOVA with two factors: time (COVID-19 pre-lockdown vs. COVID-19 post-lockdown) as a repeated measure and group (low PA, medium PA, high PA). The COVID-19 pre- and post-lockdown period runs from 15 March to 4 May 2020. Physical activity levels were classified according to pre- and post-COVID-19 METs/day difference (tertiles). Bonferroni’s post-hoc test was performed to compare changes within and between groups. All *p*-values were two-tailed with *p* < 0.05.

## 3. Results

Table 1 shows the sociodemographic characteristics of participants at baseline according to PA level. Changes in PA parameters within and between PA difference groups pre- and post-COVID-19 are shown in Table 2. There was a significant increase in terms of jump-and-reach in the high PA group (2.7 cm). After COVID-19 lockdown, participants in the high PA group had increased their jumping height (2.7 cm) more than participants in the low PA group (0.4 cm) and the medium PA group, which had worsened (−0.7 cm) after lockdown. Regarding fitness score tests, participants in the medium PA group had a worse mark (−0.1) than participants in the low PA group (0.2), who had better fitness score tests after COVID-19. The low PA group decreased sedentary activity (−48.7 min/day) and increased light (20.9 min/day) and moderate (32.3 min/day) PA intensities after lockdown. The medium PA group increased moderate PA intensity (3.5 min/day), but the high PA group showed an increase in sedentary activity (23.0 min/day) and a significant decrease in moderate PA intensity (−50.8 min/day). Between groups, the low PA group had decreased sedentary PA intensity (−48.7 min/day) and increased moderate PA intensity (32.3 min/day) in comparison with the medium PA (1.3 min/day sedentary and 3.5 min/day moderate) and high PA (23.0 min/day sedentary and −50.8 min/day moderate) groups. At the same time, the high PA group had increased sedentary activity (23.0 min/day) more than the medium PA group (1.3 min/day) and decreased moderate PA intensity (−50.8 min/day) more than the medium PA group (3.5 min/day). According to light PA intensity, participants in the high PA group decreased their level (−15.5 min/day) more than the low PA (20.9 min/day) and medium PA (2.9 min/day) groups. The medium and high PA groups decreased their sleep efficiency after lockdown (−0.8% and −1.1% respectively), whereas participants in low PA group increased their sleep efficiency (0.6%). In addition, participants in the low PA group increased their steps per day after lockdown (2479.2 steps/day), but participants in the high PA group increased more steps per day (4288.4 steps/day) than the low and medium PA (−146.8 steps/day) groups. Nevertheless, the low PA group had more steps per day than the medium PA group. Regarding MET measured by accelerometer, after lockdown, the low PA group increased 0.1 MET/day, the medium PA group obtained no difference (0.0 MET/day) and the high PA group decreased −0.2 MET/day. All of these results were significantly different between groups.

Changes in NAFLD parameters within and between PA difference groups pre- and post-COVID-19 are showed in Table 3. Participants in the low PA group had lower GGT levels (−17.0 U/L) and participants in medium and high groups higher GGT levels (12.0 U/L and 17.1 U/L respectively). Low PA was the only group who decrease GGT levels in comparison with other two. All groups had an increased FLI after lockdown (low PA, 2.5; medium PA, 5.4; high PA, 5.7) and there was a bigger in increase in the FLI of the medium PA group than the low PA group. The low and high PA groups showed a reduction in HDL-c (−2.8 mg/dL and −1.6 mg/dL, respectively). Between groups, participants in the high PA group had decreased their HDL-c levels more than the participants in the medium PA group (−0.4 mg/dL).

## 4. Discussion

The current study showed that stopping regular PA together with an unhealthy lifestyle due to COVID-19 lockdown led to a worsening of MetS and NAFLD. In fact, COVID-19 lockdown induced a PA decrease in more active people, whereas inactive people increased their PA levels, contrary to logic. As a recent systematic review stated, studies and results for adults aged 45–65 years (which is the age range of the participants in the current study) are still lacking. However, some adults increased PA during the COVID-19 pandemic, which allowed them to obtain a better fitness score test [29]. The current results are aligned with previous literature on the impact of COVID-19 lockdown on PA. Adult populations in Spain, especially very active people, decreased PA and increased sedentary activity during COVID-19 lockdown [30]. The current results showed that people in the high PA group increased levels of sedentary activity more than people in the low and/or medium PA groups. Moreover, the high PA group suffered a considerable decrease in light and moderate PA intensities. Thus, the worst results were observed among participants who performed more PA before confinement. As confirmed by previous authors, it was more common for people with high PA to suffer greater changes in regular PA than people who were not very active [31]. The current results also showed an important fact: the low PA group decreased sedentary behavior and increased light and moderate PA, which seems contradictory to most of the existing literature. Nevertheless, one study previously obtained similar results [32]. These data reflect that a good health promotion aimed at inactive people can be essential for improving their lifestyle. During lockdown, domestic activities were considered a form of PA [33,34]. PA habits changed, but motivation helped to induce changes in the PA habits of inactive people [35].

Daily METs measured by accelerometry during lockdown reflected the same patterns of behavior in each group, i.e., a decrease in daily METs by people in the high PA group compared to an in the low PA group (although it was not a very large increase). Furthermore, people in the medium PA group experienced no change in daily METs. An explanation for these data is the same as above, because a sedentary lifestyle and light and moderate PA were also measured by accelerometry. It must be pointed out that in a Chinese population, there was a sharp decline in daily step count that increased as lockdown progressed [36]. Considering that the study population in this manuscript was Spanish, the current data also showed an increase in daily step counts for both the low and high PA groups, whereas the medium PA group showed a decreased daily step count during lockdown. 

Regarding sleep efficiency, the most common trend in the population was a decrease in sleep quality due to the COVID-19 pandemic. The negative mood of lockdown has been associated with a decrease in sleep patterns, a delayed bedtime, presence of nightmares and insomnia [37,38]. Findings from this trial showed a decrease in sleep efficiency in the high and medium PA groups. This is consistent with previous literature, which showed that active people decreased PA levels and sleep quality during COVID-19 lockdown [39]. People in the low PA group increased their sleep efficiency a little, but it should be noted that they also increased PA levels during this period, which has a positive reinforcement on sleep quality [40].

After COVID-19 lockdown, people in the high PA group showed higher GGT levels, as did people in the medium PA group. Only the low PA group had decreased its GGT levels and they had the lowest levels compared to the other two groups. As already recorded, an active lifestyle based on a Mediterranean diet is associated with weight loss and improved MetS criteria in people with NAFLD. Thus, PA plays a fundamental role in the correct maintenance of GGT levels [5,41,42]. According to the current results, the high PA group decreased its active lifestyle and increased its sedentary lifestyle, whereas the low PA group increased light and moderate levels of PA and decreased their sedentary lifestyle. This event explains why the low PA group decreased GGT levels after confinement and not the high PA group.

Overall, FLI increased in all three groups, i.e., their NAFLD condition has worsened after lockdown. The low PA group had a smaller FLI increase than the medium PA group. This result is probably because the low PA group led a more active lifestyle. It is well known that steatosis status, and consequently FLI improves, when participants with NAFLD adopt a healthy lifestyle based on a Mediterranean diet and regular PA [43]. Although the low PA group had a more active lifestyle, it is shown that lockdown did not allow high enough levels of PA to improve their hepatic steatosis status.

All groups showed a decrease in HDL-cholesterol levels. This decrease was larger in the high PA group than the medium PA group. Low HDL-cholesterol level is one of the MetS parameters [44]. Regular PA can increase low HDL-cholesterol levels [44] and a Mediterranean lifestyle, with a Mediterranean diet and regular PA, is essential for improving MetS parameters [5]. It is well known that MetS is related to NAFLD, and the new tendency is to group both conditions under the one name of metabolic dysfunction-associated fatty liver disease (MAFLD) [45]. However, the COVID-19 lockdown caused an increase in the consumption of unhealthy foods and a decrease in PA levels in the population [19]. This could explain the current results, because not following a Mediterranean lifestyle intervention will worsen MetS and, in particular, low HDL-cholesterol levels.

Regarding intrahepatic fat content measured by nuclear magnetic resonance (IFC-NMR), it should be mentioned that results with significant differences were obtained with previous adjustments to the data in the current study (*p* = 0.001; data not shown). However, when socioeconomic and marital status were introduced in the current adjustment, the significance disappeared, which allows us to understand that these were two important factors during lockdown [46,47] that nullified the differences in liver fat according to PA groups.

### Strengths and Limitations of the Study

Scientific evidence for the effect of the COVID-19 lockdown on NAFLD according to PA differences is limited. The first strength of the current study is that it contributes to increasing knowledge about the impact of PA differences due to the COVID-19 lockdown in adults with NAFLD and MetS. Secondly, it considers a large range of PA and liver profile parameters, all MetS criteria, different methods of PA measurement and an alternative tool to describe functional tests with a fitness score test. Other strengths of the current work are its longitudinal design, which adds more soundness than cross-sectional designs, and the standardized protocol adopted reduces the risk of information bias. On top of these strengths, the current results show the effect of a sudden change in a healthy lifestyle to one less healthy and not complying with the recommendations of a Mediterranean diet and regular PA. Most of the existing literature shows the benefits of PA, but not the effects of stopping PA, so the COVID-19 lockdown has allowed us to study what happens when PA is stopped. However, the present work has some limitations. The main limitation is the small sample size. Other limitations are the subjectivity of the FLI, HSI and Minnesota questionnaire, even if they are validated. The information provided by participants in telephone consultations during lockdown is also subjective. The different motivation, psychological state and physical condition of the participants are other limiting factors. The participants were aged 40–60 years, which makes it difficult to extrapolate results to the general population. In addition, in a human study it is difficult to know with precision and accuracy their adherence to lifestyle treatment. Participants were asked in this regard; nevertheless, it will always be a limitation, as researchers have to trust the participants. Lastly, the study was not initially designed for this purpose, which is also a limitation.

## 5. Conclusions

Stopping regular PA together with an unhealthy lifestyle due to the COVID-19 lockdown led to a worsening of MetS and NAFLD condition. The COVID-19 lockdown induced a decrease in PA by more active people, whereas inactive people increased their PA levels. Motivation seemed to be a very important factor for the practice of PA during lockdown.

## Figures and Tables

**Table 1:** Baseline sociodemographic characteristics of study participants.

	Low PA (n = 19)	Medium PA (n = 19)	High PA (n = 19)		
	**Mean (SD)**	**Mean (SD)**	**Mean (SD)**		***p***
Age (years) ^#^	52.5 (7.7) ^a^	55.4 (5.7) ^a, c^	49.9 (6.2) ^c^		<0.001
Education (years) ^#^	14.3 (8.0)	14.6 (9.7)	13.7 (3.5)		0.454
	n (%)	n (%)	n (%)		
Gender					<0.001
Male	16 (84.2)	10 (52.6)	11 (57.9)		
Female	3 (15.8)	9 (47.4)	8 (42.1)		
Marital status					0.031
Single	3 (15.8)	1 (5.3)	2 (10.5)		
Married/coupled	14 (73.7)	13 (68.4)	12 (63.2)		
Divorced/separated/widower	2 (10.5)	5 (26.3)	5 (26.3)		
Socioeconomic status					<0.001
Low	16 (84.2)	14 (73.7)	10 (52.6)		
Medium/high	3 (15.8)	5 (26.3)	9 (47.4)		
Smoking habit					0.014
No	17 (89.5)	17 (89.5)	19 (100.0)		
≥1 cigarette/day	2 (10.5)	2 (10.5)	0 (0.0)		
Alcohol consumption					0.827
No	6 (31.6)	5 (26.3)	5 (26.3)		
<7 drinks/week	11 (57.9)	11 (57.9)	11 (57.9)		
≥7 drinks/week	2 (10.5)	3 (15.8)	3 (15.8)		

Abbreviations: PA = physical activity. Physical activity levels are classified according to pre- and post-COVID-19 METs/day difference (terciles). ^#^ = log-transformed. Differences in means between groups were tested by ANOVA. Differences in prevalence across groups were examined using χ^2^. Different letters indicate significant differences between groups (^a, c^) according to Bonferroni’s post-hoc analysis where *p* < 0.05.

**Table 2:** Changes in physical activity parameters within and between PA difference groups pre- and post-COVID-19.

		Low PA (n = 19)	Medium PA (n = 19)	High PA (n = 19)	
		Mean (SD)	Mean (SD)	Mean (SD)	T*g ^†^
Motor fitness tests					
One-leg balance (s)	Pre COVID	44.8 (19.2)	52.3 (14.2)	49.4 (17.7)	0.202
	Post COVID	49.7 (16.7)	52.7 (14.5)	53.0 (13.3)	
	Δ	4.8 (15.6) *	0.4 (10.9)	3.6 (7.4) *	
Standing hand grip (kg)	Pre COVID	44.1 (12.6)	36.8 (12.1)	37.9 (12.7)	0.335
	Post COVID	44.6 (11.8)	36.1 (10.8)	37.9 (12.7)	
	Δ	0.5 (5.8)	−0.7 (3.1) *	0.0 (5.4)	
Jump-and-reach (cm)	Pre COVID	24.2 (13.5)	23.7 (7.9)	20.9 (9.1)	0.002
	Post COVID	24.6 (9.7)	22.9 (8.6)	23.6 (6.4)	
	Δ	0.4 (12.4) ^b^	−0.7 (4.2) ^c^	2.7 (5.1) * ^b, c^	
Modified push-up (reps)	Pre COVID	9.0 (5.5)	6.8 (3.7)	7.9 (4.7)	0.502
	Post COVID	10.9 (6.4)	7.1 (5.4)	9.2 (8.1)	
	Δ	1.9 (5.3) *	0.3 (4.3)	1.3 (7.1)	
Fitness score test	Pre COVID	1.9 (1.3)	2.3 (0.9)	2.5 (1.1)	0.050
	Post COVID	2.1 (1.1)	2.2 (1.0)	2.5 (1.1)	
	Δ	0.2 (0.7) * ^a^	−0.1 (0.9) ^a^	0.0 (0.7)	
Sitting hand grip (kg)	Pre COVID	40.8 (13.1)	33.6 (13.2)	36.4 (11.6)	0.970
	Post COVID	42.1 (12.3)	34.6 (13.1)	38.2 (12.5)	
	Δ	1.3 (6.5)	1.0 (4.2) *	1.8 (5.2) *	
Chester-step (ml O_2_/kg/min)	Pre COVID	37.4 (8.8)	34.1 (7.5)	35.4 (11.3)	0.584
	Post COVID	35.2 (12.0)	33.7 (8.4)	38.2 (10.9)	
	Δ	−2.2 (10.9)	−0.4 (7.1)	2.7 (9.2) *	
Intensity PA (accelerometry)					
Sedentary (min/day)	Pre COVID	678.7 (66.0)	669.6 (103.9)	609.3 (109.7)	<0.001
	Post COVID	630.0 (83.9)	670.9 (107.0)	632.3 (105.3)	
	Δ	−48.7 (78.7) * ^a, b^	1.3 (44.6) ^a, c^	23.0 (70.4) * ^b, c^	
Light (min/day)	Pre COVID	513.9 (80.9)	489.4 (86.4)	535.3 (93.3)	<0.001
	Post COVID	534.8 (69.9)	492.3 (83.2)	519.8 (93.7)	
	Δ	20.9 (53.1) * ^b^	2.9 (47.7) ^c^	−15.5 (75.8) ^b, c^	
Moderate (min/day)	Pre COVID	166.0 (49.6)	196.7 (90.0)	233.6 (107.3)	<0.001
	Post COVID	198.3 (51.2)	200.3 (87.8)	182.8 (76.1)	
	Δ	32.3 (20.1) * ^a, b^	3.5 (13.5) * ^a, c^	−50.8 (61.1) * ^b, c^	
Sleep efficiency (%)	Pre COVID	91.5 (3.3)	92.8 (2.8)	92.5 (3.0)	<0.001
	Post COVID	92.0 (3.3)	92.0 (3.4)	91.4 (2.4)	
	Δ	0.6 (2.6) ^a, b^	−0.8 (2.0) * ^a^	−1.1 (2.3) * ^b^	
Steps (steps/day)	Pre COVID	11,779.6 (3620.6)	13,099.9 (3314.6)	15,147.8 (3359.2)	<0.001
	Post COVID	14,258.7 (3280.8)	12,953.2 (3544.5)	19,436.2 (29785.4)	
	Δ	2479.2 (2587.5) * ^a, b^	−146.8 (1221.5) ^a, c^	4288.4 (29274.4) ^b, c^	
Energy expenditure					
Measured accelerometer (MET/day)	Pre COVID	1.8 (0.2)	1.9 (0.3)	2.0 (0.3)	<0.001
	Post COVID	1.9 (0.2)	1.9 (0.3)	1.8 (0.3)	
	Δ	0.1 (0.1) * ^a, b^	0.0 (0.0) * ^a, c^	−0.2 (0.3) * ^b, c^	
Reported Minnesota (MET/day)	Pre COVID	0.7 (0.6)	0.5 (0.4)	0.5 (0.6)	0.308
	Post COVID	0.6 (0.7)	0.4 (0.5)	0.4 (0.4)	
	Δ	−0.1 (0.9)	−0.1 (0.5)	−0.1 (0.3) *	
Measured-Reported (MET/day)	Pre COVID	1.1 (0.6)	1.4 (0.4)	1.5 (0.6)	0.143

Abbreviations: Δ = change between pre- and post-COVID, cm = centimeter, kg = kilogram, MET = metabolic equivalents of task, min = minutes, ml = milliliter, No. = number, O_2_ = oxygen, PA = physical activity, reps = repetitions, s = seconds, T*g ^†^ = time*group after adjustment for age, gender, marital status and socioeconomic status according to job. Data were analyzed by two-way repeated measures ANCOVA. Different letters indicate statistically significant differences (*p* < 0.05) between time (*) time*group interaction after adjustment for age, gender, marital status and socioeconomic status according to job (^a, b, c^) by Bonferroni’s post-hoc test (*p* < 0.05). Physical activity levels are classified according to pre- and post-COVID METs/day difference (terciles). All variables were log-transformed.

**Table 3:** Changes in NAFLD parameters within and between PA difference groups pre- and post-COVID-19.

		Low PA (n = 19)	Medium PA (n = 19)	High PA (n = 19)	
		Mean (SD)	Mean (SD)	Mean (SD)	T*g ^†^
Liver profile					
AST (U/L)	Pre COVID	31.3 (23.60)	22.3 (6.6)	22.6 (6.8)	0.160
	Post COVID	25.3 (4.7)	24.9 (10.5)	30.0 (25.9)	
	Δ	−6.0 (21.5)	2.6 (9.7) *	7.4 (21.2) *	
ALT (U/L)	Pre COVID	44.6 (53.4)	27.1 (13.5)	28.3 (10.1)	0.514
	Post COVID	35.4 (18.4)	31.1 (19.8)	37.6 (34.8)	
	Δ	−9.2 (51.6)	4.0 (17.3) *	9.3 (30.2) *	
GGT (U/L)	Pre COVID	56.9 (86.9)	37.4 (16.6)	38.7 (23.5)	<0.001
	Post COVID	39.9 (28.4)	49.4 (42.1)	55.8 (62.0)	
	Δ	−17.0 (61.4) * ^a, b^	12.0 (29.6) * ^a^	17.1 (45.8) * ^b^	
FLI	Pre COVID	83.7 (13.8)	77.1 (21.7)	79.1 (21.2)	0.014
	Post COVID	86.2 (9.2)	82.5 (16.1)	84.8 (12.1)	
	Δ	2.5 (8.3) * ^a^	5.4 (22.2) * ^a^	5.7 (15.8) *	
HSI	Pre COVID	45.1 (5.6)	43.4 (4.9)	43.3 (5.0)	0.747
	Post COVID	44.2 (3.9)	43.9 (6.0)	43.5 (5.6)	
	Δ	−0.9 (4.8)	0.5 (3.1)	0.2 (2.4)	
IFC-NMR (%)	Pre COVID	13.6 (8.5)	12.7 (7.8)	12.8 (8.0)	0.119
	Post COVID	12.6 (9.2)	13.7 (9.0)	14.7 (8.4)	
	Δ	−1.0 (4.9)	1.0 (4.8)	1.9 (5.8) *	
Metabolic syndrome parameters					
Abdominal obesity (WC ≥ 94 cm men; ≥80 cm women)	Pre COVID	112.9 (9.0)	107.7 (9.4)	109.7 (7.2)	0.203
	Post COVID	112.6 (7.7)	106.4 (8.8)	110.2 (8.6)	
	Δ	−0.3 (4.4)	−1.3 (5.3) *	0.5 (4.6)	
High triglyceridemia ≥ 150 mg/dL	Pre COVID	152.7 (66.3)	193.1 (118.5)	177.4 (119.1)	0.561
	Post COVID	165.6 (62.0)	189.7 (98.9)	187.8 (121.3)	
	Δ	12.9 (55.9) *	−3.4 (78.1)	10.4 (73.1)	
Low HDL-c < 40 mg/dL men; <50 mg/dL women	Pre COVID	43.1 (9.6)	44.8 (9.3)	43.3 (10.3)	0.014
	Post COVID	40.2 (7.7)	44.4 (11.5)	41.6 (9.9)	
	Δ	−2.8 (4.9) *	−0.4 (5.4) ^c^	−1.6 (5.1) * ^c^	
Hypertension (systolic pressure ≥ 130 mmHg)	Pre COVID	130.5 (12.5)	128.1 (13.0)	131.4 (10.4)	0.718
	Post COVID	134.5 (12.7)	132.4 (17.7)	135.7 (19.1)	
	Δ	4.0 (9.5) *	4.3 (14.5) *	4.3 (16.5) *	
Hypertension (diastolic pressure ≥ 85 mmHg)	Pre COVID	80.6 (7.3)	78.9 (9.3)	79.8 (6.0)	0.282
	Post COVID	80.4 (9.2)	83.0 (13.0)	83.2 (10.1)	
	Δ	−0.2 (7.3)	4.1 (10.3) *	3.5 (9.3) *	
High fasting glycemia ≥ 100 mg/dL	Pre COVID	113.3 (36.2)	104.4 (11.3)	105.8 (31.1)	0.712

Abbreviations: Δ = change between pre and post COVID, ALT = alanine aminotransferase, AST = aspartate aminotransferase, cm = centimeter, dL = deciliter, FLI = fatty liver index, GGT = gamma glutamyl transferase, HSI = hepatic steatosis index, HDL-c = high density lipoprotein-cholesterol, IFC-NMR = intrahepatic fat content by nuclear magnetic resonance, L = liter, mg = milligram, mmHg = millimeters of mercury, NAFLD = non-alcoholic fatty liver disease, PA = physical activity, U = units, T*g ^†^ = time*group after adjustment for age, gender, marital status and socioeconomic status according job. Data analyzed by two-way repeated measures ANCOVA. Different letters indicate statistically significant differences (*p* < 0.05) between time (*) and time*group interaction after adjustment for age, gender, marital status and socioeconomic status according to job (^a, b, c^) by Bonferroni’s post-hoc test (*p* < 0.05). Physical activity levels are classified according to pre- and post-COVID METs/day difference (terciles). All variables were log-transformed.

## Data Availability

There are restrictions on the availability of data for this trial, due to the signed consent agreements around data sharing, which only allow access to external researchers for studies following the project purposes. Researchers wishing to access the trial data used in this study can make a request to pep.tur@uib.es.

## Abbreviations

ALT: alanine aminotransferase; AST: aspartate aminotransferase; BMI: Body mass index; CD: conventional diet; FLI: fatty liver index; GGT: gamma-glutamyl transferase; HDL-c: high density lipoprotein cholesterol; HSI: hepatic steatosis index; IDF: International Diabetes Federation; MET: metabolic equivalent of task; MetS: metabolic syndrome; MD-HMF: Mediterranean diet-high meal frequency; MD-PA: Mediterranean diet-physical activity; MRI: magnetic resonance imaging; NAFLD: non-alcoholic fatty liver disease; NASH: non-alcoholic steatohepatitis; PA: physical activity; SD: standard deviation; VO_2_max.: maximum oxygen volume.
